# The Relationship between Cyber Violence and Cyber Sex Crimes: Understanding the Perception of Cyber Sex Crimes as Systemic Issues

**DOI:** 10.3390/children11060682

**Published:** 2024-06-04

**Authors:** Eugene Lee, Hye Eun Lee

**Affiliations:** 1 Annenberg School for Communication and Journalism, University of Southern California, University Park Campus, Los Angeles, CA 90089, USA; elee9487@usc.edu; 2 Department of Communication & Media, Ewha Womans University, Seoul 03760, Republic of Korea

**Keywords:** cyber sex crime, cyber violence, teacher intervention, victim, perpetrator, systemic

## Abstract

This study examines the relationship between cyber violence and cyber sex crimes, specifically focusing on these crimes as systemic issues among adolescents. The research highlights the severe impact of cyber sex crimes, characterized by the non-consensual sharing of sexually explicit content. It examines various factors that may contribute to witnessing cyber sex crimes, including exposure to violent online content, personal experiences of cyber violence (either as a victim or perpetrator), and the role of parental and teacher interventions. Utilizing data from a nationwide survey conducted by the Korea Communications Commission, the study analyzes responses from 9016 adolescents in 2021 and 9693 in 2022. This analysis reveals significant predictors of witnessing cyber sex crimes and examines how perceptions of cyber violence and interventions of authoritative figures may influence adolescents’ perception of cyber sex crimes as either systemic or individual issues. With females disproportionately affected, the findings underscore a gendered aspect of cyber violence. Furthermore, these insights suggest that perceiving cyber violence as a serious issue leads to viewing cyber sex crimes as systemic problems necessitating societal intervention. The study advocates for enhanced digital literacy education and systemic changes to protect adolescents from the widespread threats of cyber violence and sex crimes.

## 1. Introduction

In the contemporary digital landscape, cyber sex crimes against adolescents represent a growing area of concern. Cyber sex crimes are defined as actions involving the unauthorized use of cameras (including those in smartphones) to secretly record and distribute intimate images of individuals without their consent, as well as engaging in sexual harassment within online environments. This includes the following: covertly recording individuals’ bodies without their permission, sharing content that may cause sexual embarrassment through digital platforms without consent, manipulating and distributing sexually explicit versions of publicly shared photos along with personal information, luring victims via online platforms to groom them for sexual exploitation, and threatening to distribute recordings of obscene activities during video chats. A pivotal study indicates that before the age of 18, 16% of young adults in the US had experienced some form of online sexual abuse [1]. The national survey study underscores the diversity of perpetrators, including known adults and peers who engage in acts such as the nonconsensual misuse of sexual images, “sextortion”, and online grooming.

Cyber sex crimes, a critical subset of the broader phenomenon of cyber violence, pose a significant threat in the digital world, especially to adolescents who misuse and abuse new media [2]. These crimes specifically entail the unauthorized capture and sharing of sexually explicit content without the consent of those depicted; this distinguishes them from other forms of cyber violence, such as cyberbullying, threats, and the distribution of sensitive non-sexual content. The literature suggests that widespread exposure to violent and aggressive media content may desensitize individuals to the gravity of these actions [3], potentially increasing the likelihood of both perpetrating and becoming a victim of cyber sex crimes. This desensitization underscores a troubling trend where the normalization of aggression through digital media contributes to an environment ripe for such offenses. Adolescents, who are particularly active online, find themselves at an increased risk, not just of cyber violence in general, but of cyber sex crimes more specifically.

The purpose of this study is to examine the factors that may lead to witnessing cyber sex crime. Specifically, it delves into the following key areas: the degree of exposure to violent online content, personal experiences of cyber violence as either a victim or perpetrator, the impact of parental and teacher interventions, awareness of effective strategies for reporting cyber violence and understanding the legal ramifications associated with such acts. Furthermore, this study examines whether the way that adolescents experience and understand cyber violence shapes how they perceive cyber sex crime and investigates whether adolescents regard these incidents as systemic problems or as matters of individual concern.

Awareness of cyber sex crimes in South Korea has risen sharply due to the following two high-profile cases: Welcome to Video (W2V) and the Nth Room [4]. Both were operated by South Korean nationals, the former was the largest known child pornography site, while the latter sold videos of forced acts on young women and children. Despite legal action and seizures, the number of reported digital sex crimes continues to rise [4]. South Korea is highly digitized, with one of the highest rates of internet penetration and smartphone use in the world [5]. This digital saturation influences social interactions, particularly among adolescents, who are the primary users of digital media and platforms in the country. As sex crimes increasingly move online and take advantage of digital platforms, adolescents are more likely to be exposed to inappropriate content and predatory behavior.

Gender dynamics in South Korea also contribute to the complexity of addressing cyber sex crimes. The country has traditional gender roles that can affect the perception and reporting of cyber violence. Women and girls often face significant social stigma when victimized online, which can deter the reporting of such crimes and reduce the efficacy of interventions. By providing this context, our study aims to shed light on the systemic issues that perpetuate cyber sex crimes in South Korea and underscore the need for tailored interventions that take into account these unique cultural and digital contexts. This approach not only enriches our understanding of the situation but also guides more effective policy and educational strategies to combat cyber sex crimes among South Korean adolescents.

### 1.1. Cyber Sex Crime and Cyber Violence

Cyber sex crime is described as an intentional act that leverages digital technology to control, shame, and humiliate individuals, often in a sexual context. This includes non-consensual sharing of intimate images, sexual harassment, and the use of digital platforms to stalk or blackmail individuals [6].

Addressing cyber sex crimes is crucial due to their enduring impact on adolescents, often precipitating mental health challenges. Recent investigations have highlighted text messaging and social media platforms such as Facebook, Instagram, Snapchat as common realms where adolescents frequently encounter sexual harassment [7,8,9,10,11].

A substantial number of adolescent girls have encountered various forms of cyber sexual harassment, revealing significant correlations with substance abuse, mental health complications, and histories of sexually transmitted infections (STIs) [12]. This suggests that cyber sexual harassment intersects with broader health issues among adolescents, underscoring the multifaceted repercussions of such experiences [12].

In previous research, cyber sex crime affects a significant number of emerging young women, endangering their offline personal privacy and safety, and this necessitates more public education and legal changes to protect victims [6]. However, schools and institutions lack the resources to offer adequate support, underscoring the need for systemic changes.

### 1.2. Increased Use of Online Interaction and Exposure to Violent Media Content

The proliferation of online platforms has become a central aspect of communication among today’s youth, who are driven by technological advancements. Data show that 95% of U.S. adolescents own some form of mobile device, with 89% specifically owning smartphones [13,14]. Similarly, in South Korea, the early adoption of smartphones among youth, with 92.6% of sixth graders reporting ownership, mirrors this global trend. This surge in digital interaction and social media engagement has led to new variants of aggression, including cyberbullying, harassment, unauthorized sharing of intimate images, and further exposure to cyber sex crimes, with significant implications for adolescent communities [15,16].

Expanding on these concerns, the integration of information and communication technologies (ICTs) into the daily lives of adolescents not only enables but also exacerbates vulnerability to cyber sexual violence. Research points to the tendency of adolescents to engage in dangerous online activities such as sexting, highlighting the urgent need for comprehensive digital literacy education and online safety measures to counter these emerging threats [17]. Research shows that online chat room interactions between adults and minors often evolve into sexual relationships [18]. Significantly, 75% of these cases involved girls between the ages of 13 and 15, and 76% of the perpetrators were over the age of 25. Surprisingly, these adults usually did not hide their age or their intent to engage in sexual relations. Many victims had repeated encounters with these adults, with about half reporting deep emotional attachments or feelings of love for their perpetrators. Where male victims were involved, almost all perpetrators were male. Violence was rare, although present in a few cases, and indicated sporadic use of coercion or force.

Widespread ownership of personal devices complicates guardian supervision of digital content, increasing the risk of exposure to aggressive online behaviors and content among the younger populations [19]. In this context, South Korea presents an interesting case study, as younger generations begin to use smartphones at an early age, providing a unique opportunity to closely investigate the risks of digital exposure among this younger population.

### 1.3. Cyber Victimization, Perpetration, and Exposure to Cyber Sex Crime

Previous studies indicate that exposure to cyber violence, whether as a victim or perpetrator, may increase susceptibility to cyber sex crimes, highlighting the intertwined nature of these online dangers. For instance, cyber victimization and psychological intimate partner violence contribute to depressive symptoms among college freshmen, with cyber victimization uniquely linked to antisocial behavior. This suggests that the experience of cyber violence may have lasting emotional and behavioral impacts, potentially increasing vulnerability to further online exploitation [20]. Regular exposure to cyber violence could lead to its normalization among adolescents, resulting in a diminished response to witnessing online violence [2]. Previous studies have mentioned the blurred lines between victims and perpetrators in the context of cyber violence [3]. Victims and perpetrators of cyberbullying often overlap, and individuals with lower levels of self-control are more likely to engage in cyberbullying behaviors after being cyberbullied themselves [21], potentially leading to a higher risk of encountering cyber sex crimes.

In this study, we suggest that both exposure to violent media content and experiences as a victim and perpetrator will have a positive relationship with witnessing cyber sex crimes. Adolescents exposed to dangerous environments are more susceptible to encountering illegal activities such as cyber sex crimes. The link between experiencing cyber violence in real life and its continuation in online spaces deserves close attention. For example, a previous study that investigated intimate partner violence victimization in both cyber and physical domains found that victims of cyber aggression often experience concurrent in-person psychological, physical, and sexual partner violence [22]. This finding suggests that incidents of cyber violence are not isolated, but are part of a broader spectrum of abusive behaviors that may include cyber sex crimes.

In this study, we argue that adolescents who have more exposure to violent media content are more likely to witness cyber sex crimes online because they are not in a safe environment and may have normalized witnessing such actions. We also believe that participants who have previous experience in cyber violence, whether as victims or perpetrators, will also be more likely to witness cyber sex crimes because they are exposed to unsafe environments.

**H1.**  Exposure to violent media content will have a positive relationship with witnessing cyber sex crimes.

**H2.**  Experiences of being a victim (H2a) and a perpetrator of cyber violence (H2b) will have a positive relationship with witnessing cyber sex crimes.

**H2a.**  Experiences of being a victim.

**H2b.**  A perpetrator of cyber violence.

### 1.4. Parent and Teacher Intervention and Knowing Viable Responses and Legal Consequences

Previous studies have addressed the role of social–interpersonal factors in explaining cyber violence victimization and perpetration. One study found that anxious attachment and lack of social support increased the likelihood of various forms of cyber dating abuse, suggesting that emotional and social vulnerabilities can play a significant role in the risk of experiencing cyber sex crimes [23].

Addressing cyber sex crime effectively demands a dual approach that encompasses both preventative strategies and reactive solutions [24]. The cornerstone of such efforts is digital literacy, which involves the competency to decipher and utilize information gleaned from various online platforms [25]. This skill set is indispensable for evaluating the credibility of digital content and steering clear of online environments that may pose risks [26]. Research has underscored that a deficiency in digital literacy may precipitate issues such as diminished self-regulation [27] and susceptibility to online addiction [28]; each of which can escalate the likelihood of engaging in problematic behaviors on the internet and encountering cyber violence, which may lead to exposure to cyber sex crimes.

The educational influence of parents and teachers is crucial in equipping adolescents with the skills to navigate and counteract cyber violence. Through proactive engagement, guardians can significantly reduce the negative impact that media can have on young individuals [29,30]. By imparting knowledge about safe online practices and advising on cautious interaction with digital content and individuals online, parents and teachers play a key role in enhancing digital literacy among the youth and preventing cyber sex crimes against them.

Moreover, awareness of appropriate actions to take after experiencing or witnessing cyber violence is vital for reducing its occurrence [6,24]. Incorporating values and moral education into the curriculum can promote appropriate behavioral responses following encounters with cyber violence. Such educational endeavors aim to instill and cultivate values that advocate for positive conduct [31]. For instance, educating adolescents about the importance of protecting their peers from cyber violence and providing them with practical steps to take, such as reporting such incidents, are essential interventions. According to a previous study, knowing the legal consequences of cyber crime can help reduce this behavior [32]. This research aims to explore how parental and teacher guidance, along with an understanding of effective response mechanisms, can reduce the number of adolescents encountering cyber sex crimes.

### 1.5. Gender and Exposure to Cyber Sex Crimes

Research sheds light on how social media platforms and digital communication tools like Facebook, Instagram, Snapchat, and text messaging, are becoming venues for sexual harassment, particularly among adolescents. Studies show that cyber violence often takes on gendered dimensions, disproportionately affecting women, girls, and sexual and gender minorities [33]. This study examines the growth of information and communication technologies and how they have facilitated gendered violence, with women, girls, and sexual and gender minorities being the main targets. Emerging young women are particularly at risk of cyber sexual violence, which includes various forms of online gender and sexual exploitation that can lead to significant mental health consequences [6]. A previous study pointed out that cyber sex crimes occur primarily among teenage girls between the ages of 13 and 15 who are exposed to adult perpetrators they meet through online chat platforms [18].

The feminist epistemology of digital crime encourages a thorough examination of gender issues in various aspects of the digital space, including crime and justice. This approach explicitly acknowledges power differentials between males and females and integrates this understanding into the analysis of crime and gender relations in cyberspace [34]. The framework illuminates intersectionality by demonstrating how various social axes, such as gender, sexuality, and age, intersect to shape an individual’s experiences of cyber violence [34,35]. According to the framework, perceptions of cybercrime may differ by gender [34], which could lead to differential exposure to cyber sex crimes. For example, a previous study investigated gender differences in perceptions of cybercrime and found that while women in the United Kingdom perceive psychosocial cybercrimes (e.g., cyberbullying, revenge porn) as more serious than men, there were no significant gender differences in perceptions of socio-economic cybercrime like online fraud, highlighting the gendered nature of psychosocial versus socio-economic cybercrimes [36]. Since our study focuses on cyber sex crimes, which are a type of psychosocial cybercrime, we can argue that the experiences surrounding cyber sex crimes will differ based on gender. As South Korea still adheres to hierarchical gender norms, an examination of power dynamics reveals how patriarchal structures can contribute to the normalization of violence against women and marginalized genders. This normalization plays out in digital spaces, reflecting offline gender power imbalances that sanction or ignore such behaviors [34,35]. To account for gender, we hypothesize the following:

**H3.**  Female adolescents will be more likely to witness cyber sex crimes compared to male adolescents.

### 1.6. Perceiving Cyber Sex Crime as a Systemic Issue vs. an Individual Issue

The literature on the perceptions of cyber sex crimes offers diverse viewpoints, highlighting the multifaceted nature of this issue. These perspectives range from viewing cyber sex crimes as systemic problems, facilitated by the anonymity of online media and inadequate legal consequences, to understanding them as the result of individual motives, such as financial gain, a desire for attention, or a lack of seriousness regarding the impact of such crimes.

Previous studies have discussed the critical role of perceptions of punishment in reporting intentions, emphasizing that the certainty of punishment significantly influences the likelihood of reporting cyber sexual violence crimes. This suggests that enhancing the visibility and certainty of punishment could be a key factor in combating cyber sex crimes, indicating a systemic issue in which the effectiveness of legal frameworks and their enforcement play a crucial role [37].

Another study discussed the causes of cyber crimes, including cyber sex crimes, and highlighted prevention methods. The findings indicated that cyber sex crimes occur due to a combination of factors, including technological advancements, lack of security measures, and the lure of easy financial gain or other benefits from such crimes, indicating both systemic and individual factors that contribute to the prevalence of cyber sex crimes [38].

A previous study examined the mental health impacts of cyber sexual violence on young women, indicating that schools and institutions often lack the resources and knowledge to effectively address and prevent cyber sexual violence [6]. This underscores systemic issues related to education and institutional preparedness in addressing cyber sex crimes, as well as the need for comprehensive public education and legal reforms to protect individuals from such violence [6].

In this study, we hypothesize that perceptions of cyber violence will influence adolescents’ views of cyber sex crimes. If adolescents understand the severity of cyber violence, they are likely to recognize the importance of intervening in cyber sex crimes and perceive them as societal and systemic issues rather than merely individual matters. Furthermore, we argue that participants who have had more guidance from parents and teachers in understanding media usage and cyber violence are more likely to view the recurrence of cyber sex crime as a systemic problem rather than an individual problem.

**H4.**  Perceptions of cyber violence will influence perceptions of cyber sex crime. Specifically, adolescents who perceive cyber violence as a serious problem will view cyber sex crime as a systemic issue rather than a matter of individual motives.

**H5.**  Adolescents who have received intervention from parents (H5a) and teachers (H5b) will view cyber sex crimes as a systemic issue rather than as matters of individual motives.

**H5a.**  Adolescents who have received intervention from parents.

**H5b.**  Adolescents who have received intervention from teachers.

## 2. Materials and Methods

The data were obtained from an annual nationwide survey of South Korean adolescents carried out by the Korea Communications Commission (KCC), which is an organization endorsed by the presidency to regulate communications, from 2021 to 2022. This survey focused on the issue of cyber violence among teenagers, with the goal of understanding the challenges faced in Korea, since it began in 2013. The responsibility for conducting these surveys was given to the National Information Society Agency, which was founded in 1987 under the auspices of Korea’s Ministry of Science and ICT and the Ministry of the Interior and Security.

### 2.1. Data Collection

Data collection involved using stratified random sampling, selecting whole classes by regions and grades from the School Statistics database managed by the Korea Educational Development Institute. Teachers supervised students as they completed the survey, and participation was mandatory. This resulted in a 100% participation rate, although some questions may not have been answered. The data covered South Korean students from the 4th grade of elementary school through the last year of high school, with the country’s academic system spanning six years of elementary school, three years of middle school, and three years of high school. Typically, elementary school 4th graders are about 10 years old, while high school seniors are approximately 18 years old.

The following two separate analyses were conducted: (1) identifying factors that lead to witnessing cyber sex crime, and (2) investigating specific factors that may affect whether adolescents view the ongoing prevalence of cyber sex crime as resulting from systemic problems or individual actions (*N*_2021_ = 9016, *N*_2022_ = 9693). We eliminated four outliers based on the box plot. Specifically, these were individuals marked as having experienced both victimization and perpetration across all categories of cyber violence.

### 2.2. Measurements

#### 2.2.1. Witnessing Cyber Sex Crimes

Participants were asked to report how often they had witnessed the following types of cyber sex crimes. The following five different types of cyber sex crimes were included: (1) footage taken secretly without consent using a digital camera or smartphone camera, (2) footage that causes sexual embarrassment to others and is distributed online without consent, such as through the internet, (3) footage maliciously edited using images from social media to include sexual content and then distributed along with personal information, (4) situations or content in which the victim is lured through online chat, mobile messengers, SNS, etc., groomed to facilitate sexual acts or similar behavior, and to prevent the exposure of harm; (5) footage of obscene acts recorded through a chat application. If participants have witnessed any cases of cyber sex crimes, they were coded as 1, and if they did not witness any cyber sex crimes, they were coded as 0.

#### 2.2.2. Perception of Cyber Sex Crime

Perceptions of cyber sex crimes mean understanding whether individuals attribute the recurrence of such crimes to systemic or individual factors. Participants were asked to select one of five reasons to explain the continued occurrence of cyber sex crimes. Systemic issues included the lack of severe punishment and the anonymity provided by the internet, which makes it difficult to apprehend perpetrators. This perspective was coded as 1. Conversely, attributions to individual motivations such as financial gain, seeking online attention, or underestimating the seriousness of these crimes were coded as 0.

#### 2.2.3. Grade

The survey included participants ranging from 4th grade in elementary school up to 3rd year in high school. To reflect the South Korean education system, which consists of three years of middle school and three years of high school, the grades were coded numerically as follows: 4th grade in elementary school as 1, 5th grade as 2, 6th grade as 3, 1st year in middle school as 4, 2nd year as 5, 3rd year as 6, 1st year in high school as 7, 2nd year as 8, and 3rd year as 9.

#### 2.2.4. Gender

Males were coded as 0, while females were coded as 1.

#### 2.2.5. Average Internet Usage

Average daily internet use was measured on a 6-point scale (0 = less than 1 h, 1 = 1–2 h(s), 2 = 2–3 h, 3 = 3–4 h, 5 = 4–5 h, and 6 = 5 or more hours).

#### 2.2.6. Exposure to Violent Media Content

Participants were asked to report how often they had seen the following types of content (text, cartoon, picture, video, etc.) online (including pop-up windows). The following five categories were included: violent content, sensational content depicting male/female body parts or sexual interactions, derogatory comments about celebrities and politicians, content involving illegal behavior (fraud, theft, etc.), and false information presented as fact. A 5-point response scale was used (0 = *none*, 1 = *once or twice a year*, 2 = *once or twice in a few months*, 3 = *once or twice a month*, 4 = *once or twice a week*).

In 2021, the factor comprised five items that explained 72.79% of the variance with factor loadings from 0.89 to 0.81 (α = 0.91). In 2022, the factor comprised five items that explained 65.51% of the variance with factor loadings from 0.73 to 0.85 (α = 0.87).

#### 2.2.7. Experience of Being a Victim

Six items were used to measure whether or not participants had been victims of cyber violence. The instruction question was as follows: “In the past year, have you experienced any of the following types of cyber violence while using the internet/smartphone? If so, how often did you experience it?” The six types of cyber violence listed were as follows: “Someone made derogatory comments about me or hurt my feelings”, “Someone made a false rumor or spread an exaggerated story about me”, “Someone kept sending emails/messages or posted pictures or messages on my blog and SNS knowing that I did not like them”, “Someone sent sensational pictures or videos without my consent knowing that I did not like them”, “Someone posted my personal information (name, address, school name, photos, etc.) without my consent”, and “Someone prevented me from leaving the chat room and made fun of me or made derogatory comments about me and did not let me participate in the conversation”. If participants experienced any category of victimization, it was coded as 1, and if they did not experience any victimization, it was coded as 0.

#### 2.2.8. Experience of Being a Perpetrator

Six items were used to measure whether or not participants had been perpetrators of cyber violence. The instruction question was as follows: “In the past year, have you done any of the following things using the internet/smartphone? If so, how often did you do it?” The following six items were asked: “I have made derogatory comments or hurt other people’s feelings”, “I have spread false rumors or exaggerated stories about other people”, “I have repeatedly sent emails/messages or posted pictures or messages on other people’s blogs and SNS knowing that other people do not like it”, “I have sent sensational pictures or videos without consent knowing that other people do not like it”, “I have spread other people’s personal information (name, address, school name, photos, etc.) without their consent”, and “I stopped someone from leaving the chat room and made fun of them or made derogatory comments and did not let them join the conversation”. If participants committed any category of offense, it was coded as 1, and if they did not commit any category of offense, it was coded as 0.

#### 2.2.9. Perception of Cyber Violence

Perceptions of cyber violence were assessed based on how problematic participants perceived cyber violence to be. The items that were used to evaluate participants’ perceptions were eight categories of cyber violence such as ‘making derogatory comments or hurting other people’s feelings’ and ‘spreading false rumors or exaggerating stories about others’. A 4-point response scale was used (1 = *not at all problematic*, 2 = *not problematic*, 3 = *problematic*, 4 = *very problematic*).

In 2021, the factor consisted of eight items that explained 73.22% of the variance with factor loadings ranging from 0.78 to 0.89 (α = 0.95). In 2022, the factor included eight items that explained 79.43% of the variance with factor loadings ranging from 0.78 to 0.92 (α = 0.96).

#### 2.2.10. Parental Intervention

Parental intervention refers to the question “How much does your parent intervene when you use the internet/smartphone?” The scale included the following four safety interventions: (1) mediating with monitoring software, (2) educating adolescents about inappropriate websites, programs, and apps, (3) educating adolescents about what not to share online, and (4) regulating adolescents’ internet and smartphone use time. Participants were asked to indicate how many of these supervision methods apply to them. The sum of the items ranged from 0 to 4, with higher scores indicating more parental intervention.

#### 2.2.11. Teacher Intervention

Teacher intervention was measured by asking “How much does the school or teacher intervene with internet/smartphone use and cyber violence?” Items included (1) our school has rules about cyber violence, (2) our school prevents students from using smartphones during class, (3) teachers at our school often talk about cyber violence, and (4) our school has had preventive education about cyber violence. Participants were asked to indicate how many of these apply to them. The sum of the items ranged from 0 to 4, with higher scores indicating more teacher intervention.

#### 2.2.12. Knowing Viable Responses to Cyber Violence

A viable response to cyber violence refers to knowing what to do if a participant witnesses cyber violence. In this study, we operationalized a viable response as knowing the website or number to report cyber violence. If participants knew, it was coded as 1, and if they did not know, it was coded as 0.

#### 2.2.13. Knowing the Legal Consequences of Cyber Violence

Participants were asked if they were aware that cyber violence can have legal consequences. If they were aware, it was coded as 1; if they were not aware, it was coded as 0.

#### 2.2.14. Cyber Violence Education Necessity

Participants were asked whether they thought that cyber violence was necessary using an item. A 4-point response scale was used (1 = *not at all necessary*, 2 = *not necessary*, 3 = *somewhat necessary*, 4 = *very necessary*).

## 3. Results

### 3.1. Logistic Regression on Witnessing Cyber Sex Crimes

Because one of our key variables was gender, a chi-squared test of independence was conducted to examine the relationship between gender and experience of witnessing cyber sex crime. The relationship between these variables was not significant in 2021, *X*^2^ (1, *N* = 9016) = 0.85, *p* = 0.36, but was significant in 2022, *X*^2^ (1, *N* = 9689) = 8.78, *p* = 0.003. In 2022, females were more likely than males to witness cyber sex crime.

Regarding the relationship between gender and perceiving cyber sex crime as a systemic issue, the relationship between these variables was significant in 2021, *X*^2^ (1, *N* = 9016) = 40.17, *p* < 0.001, and was also significant in 2022, *X*^2^ (1, *N* = 9689) = 99.31, *p* < 0.001. The correlation table is available in the Appendix A.

Logistic regression was conducted to investigate the effects of independent variables (i.e., grade, gender, average internet usage, exposure to violent media content, experience of being a victim, experience of being a perpetrator, perceptions of cyber violence, parental intervention, teacher intervention, knowledge of viable responses to cyber violence, and knowledge of legal consequences of cyber violence) on the likelihood of witnessing cyber sex crimes versus not being exposed to cyber sex crimes (see Table 1). The logistic regression models were statistically significant in 2021 (*X*^2^ (11, *N* = 9016) = 483.77, *p* < 0.001), and in 2022 (*X*^2^ (11, *N* = 9689) = 759.3, *p* < 0.001). For 2021, the model explained 11.5% and for 2022, the model explained 15.6% of the variance in explaining witnessing cyber sex crimes.

According to the 2021 model, grade level, gender, exposure to violent media content, experience of being a victim, experience of being a perpetrator, and teacher intervention were significant factors that were related to witnessing cyber sex crimes. For grade (*B* = 0.06, *SE* = 0.02, *Wald* = 15.24, *p* < 0.001), the estimated odds ratio favored an increase of approximately 6% (*Exp*(*B*) = 1.06, 95% CI (1.03, 1.10)) for each unit increase in grade. Furthermore, gender (*B* = 0.16, *SE* = 0.08, *Wald* = 4.43, *p* = 0.04, *Exp*(*B*) = 1.17, 95% CI (1.01, 1.36)), exposure to violent media content (*B* = 0.15, *SE* = 0.01, *Wald* = 233.9, *p* < 0.001, *Exp*(*B*) = 1.16, 95% CI (1.14, 1.19)), experience of being a victim (*B* = 0.36, *SE* = 0.09, *Wald* = 16.78, *p* < 0.001, *Exp*(*B*) = 1.43, 95% CI (1.2, 1.69)), experience of being a perpetrator (*B* = 0.80, *SE* = 0.09, *Wald* = 71.71, *p* < 0.001, *Exp*(*B*) = 2.22, 95% CI (1.84, 2.66)), and teacher intervention (*B* = 0.08, *SE* = 0.03, *Wald* = 8.65, *p* < 0.001, *Exp*(*B*) = 1.09, 95% CI (1.03, 1.15)) were significant. In 2021, adolescents who were in higher grades, females, those who were more exposed to violent media content, those with experiences of being a victim or perpetrator, and those who had more teacher intervention witnessed more cyber sex crimes. Hypotheses on the relationship between exposure to violent media (H1), experience of being a victim (H2a), experience of being a perpetrator of cyber violence (H2b), and being female (H3) were all supported, positively associating with witnessing cyber sex crimes.

According to the 2022 model, gender, exposure to violent media content, experience of being a victim, experience of being a perpetrator, and perception of cyber violence were related to witnessing cyber sex crimes. To be specific, gender (*B* = 0.39, *SE* = 0.08, *Wald* = 27.91, *p* < 0.001, *Exp*(*B*) = 1.48, 95% CI (1.28, 1.72)), exposure to violent media content (*B* = 0.18, *SE* = 0.01, *Wald* = 363.7, *p* < 0.001, *Exp*(*B*) = 1.2, 95% CI (1.17, 1.22)), experience of being a victim (*B* = 0.43, *SE* = 0.08, *Wald* = 28.19, *p* < 0.001, *Exp*(*B*) = 1.54, 95% CI (1.31, 1.81)), experience of being a perpetrator (*B* = 0.67, *SE* = 0.09, *Wald* = 61.25, *p* < 0.001, *Exp*(*B*) = 1.96, 95% CI (1.67, 2.33)), and perception of cyber violence (*B* = −0.23, *SE* = 0.07, *Wald* = 12.36, *p* < 0.001, *Exp*(*B*) = 0.79, 95% CI (0.70, 0.90)) were significant. In 2022, adolescents who were females, those who were more exposed to violent media content, those with experiences of being a victim or perpetrator, and those who did not consider cyber violence to be problematic witnessed more cyber sex crimes. Hypotheses on the relationship between exposure to violent media (H1), experience of being a victim (H2a), and experience of being a perpetrator of cyber violence (H2b) and being female (H3) were all supported in 2022 as well, showing a consistent pattern between 2021 and 2022.

### 3.2. Logistic Regression on Perceiving Cyber Sex Crime as a Systemic Issue

Logistic regression was conducted to investigate the effects of independent variables (i.e., grade, gender, average internet usage, exposure to violent media content, experience of being a victim, experience of being a perpetrator, perceptions of cyber violence, parental intervention, teacher intervention, knowledge of viable responses to cyber violence, knowledge of legal consequences of cyber violence, and witnessing cyber sex crimes) on the likelihood of predicting perceiving cyber sex crime as a systemic issue (see Table 2). The logistic regression models were statistically significant in 2021 (*X*^2^ (12, *N* = 9016) = 267.35, *p* < 0.001), and in 2022 (*X*^2^ (12, *N* = 9689) = 175.47, *p* < 0.001). For 2021, the model explained 3.9% and for 2022, the model explained 2.4% of the variance in explaining the perception of cyber sex crimes.

According to the 2021 model, grade level, gender, average internet usage, exposure to violent media content, experience of being a perpetrator, perception toward cyber violence, teacher intervention, knowing legal consequences, and thinking cyber violence education is necessary were significant factors that were related to perceiving cyber sex crime as a systemic issue. For grade (*B* = 0.04, *SE* = 0.01, *Wald* = 19.14, *p* < 0.001), the estimated odds ratio favored an increase of approximately 4% (*Exp*(*B*) = 1.04, 95% CI (1.02, 1.06)) for each unit increase in grade. Additionally, gender (*B* = 0.23, *SE* = 0.04, *Wald* = 27.41, *p* < 0.001, *Exp*(*B*) = 1.26, 95% CI (1.15, 1.37)), average internet usage (*B* = 0.09, *SE* = 0.02, *Wald* = 38.07, *p* < 0.001, *Exp*(*B*) = 1.1, 95% CI (1.06, 1.13)), exposure to violent media content (*B* = −0.02, *SE* = 0.01, *Wald* = 19.08, *p* < 0.001, *Exp*(*B*) = 0.98, 95% CI (0.97, 0.99)), experience of being a perpetrator (*B* = −0.14, *SE* = 0.07, *Wald* = 3.84, *p* = 0.049, *Exp*(*B*) = 0.87, 95% CI (0.76, 1.00)), perception toward cyber violence (*B* = 0.33, *SE* = 0.05, *Wald* = 54.11, *p* < 0.001, *Exp*(*B*) = 1.39, 95% CI (1.27, 1.52)), teacher intervention (*B* = 0.03, *SE* = 0.02, *Wald* = 4.41, *p* = 0.04, *Exp*(*B*) = 1.03, 95% CI (1.00, 1.07)), knowing legal consequences (*B* = −0.16, *SE* = 0.05, *Wald* = 9.99, *p* < 0.001, *Exp*(*B*) = 0.85, 95% CI (0.77, 0.94)), and thinking cyber violence education is necessary (*B* = 0.15, *SE* = 0.04, *Wald* = 17.68, *p* < 0.001, *Exp*(*B*) = 1.16, 95% CI (1.08, 1.25)) were significant.

In 2021, adolescents who were in higher grades, females, those who spent more time online, those who were less exposed to violent media content, those who did not have experience of being a perpetrator, those who considered cyber violence problematic, those who had more teacher intervention, those who were not aware of legal consequences, and those who thought cyber violence education was necessary considered cyber sex crimes to stem from systemic reasons rather than from individual motivation. In our study, we hypothesized that adolescents who perceive cyber violence as problematic will likely view cyber sex crime as a systemic issue rather than considering it to stem from individual motives (H4), and the result supported H4. We also hypothesized that adolescents who had parent intervention (H5a) and teacher intervention (H5b) would consider cyber sex crime as a systemic issue. While H5b was supported, H5a was not supported.

According to the 2022 model, grade level, gender, perception toward cyber violence, teacher intervention, and thinking cyber violence education is necessary were significant factors that were related to perceiving cyber sex crime as a systemic issue. For grade (*B* = 0.04, *SE* = 0.01, *Wald* = 18.38, *p* < 0.001), the estimated odds ratio favored an increase of approximately 4% (*Exp*(*B*) = 1.04, 95% CI (1.02, 1.06)) for each unit increase in grade. Similarly, gender (*B* = 0.35, *SE* = 0.04, *Wald* = 66.51, *p* < 0.001, *Exp*(*B*) = 1.42, 95% CI (1.30, 1.54)), perception toward cyber violence (*B* = 0.14, *SE* = 0.04, *Wald* = 12.38, *p* < 0.001, *Exp*(*B*) = 1.14, 95% CI (1.06, 1.23)), and teacher intervention (*B* = 0.03, *SE* = 0.01, *Wald* = 5.26, *p* < 0.001, *Exp*(*B*) = 1.03, 95% CI (1.01, 1.06)), and thinking cyber violence education is necessary (*B* = 0.12, *SE* = 0.03, *Wald* = 14.92, *p* < 0.001, *Exp*(*B*) = 1.12, 95% CI (1.06, 1.19)) were significant.

In 2022, adolescents who were in higher grades, females, those who considered cyber violence problematic, those who had more teacher intervention, and those who thought that education on cyber violence was necessary considered cyber sex crimes to stem from systemic reasons rather than from individual motivations. The 2022 model followed the 2021 model in terms of the relations between perception toward cyber violence and perceiving cyber sex crimes as a systemic issue, supporting H4. Similarly, it did not support H5a but supported H5b, showing significance in teacher intervention but not parent intervention.

## 4. Discussion

This study aimed to explore the factors that contribute to the observation of cyber sex crimes among adolescents and to determine whether these incidents are perceived as resulting from systemic problems or from individual motivations.

### 4.1. Widespread Exposure and the Need for Targeted Interventions

Our findings suggest that approximately 9 to 10 percent of adolescents witnessed cyber sex crimes in 2021 and 2022, respectively, highlighting a significant risk across digital platforms. Given the clear legal implications of such crimes, the high rate of exposure among adolescents underscores the urgency of targeted interventions to protect young people, particularly through their frequent online engagements.

### 4.2. The Risk of Desensitization to Cyber Violence

The study reveals a concerning link between exposure to violent media content and witnessing cyber sex crimes. Adolescents who are immersed in a violent media environment and who are exposed to cyber victimization are at increased risk of encountering online sexual crime content. This exposure risks desensitizing them to the seriousness of such acts, potentially increasing the likelihood of becoming both a perpetrator and a victim of cyber sex crimes. These findings underscore the essential role of digital literacy programs offered by schools and institutions in fostering critical thinking and promoting ethical behavior online.

### 4.3. Gendered Nature of Cyber Violence

Our findings show that females are disproportionately exposed to cyber sex crime content, highlighting the need for gender-specific strategies that address the unique vulnerabilities faced by young women and girls [33]. This includes raising awareness of gender-based violence in digital spaces and promoting equality and respect in online interactions. The intersectional approach from the feminist epistemology of digital crime illuminates how these vulnerabilities are not only gendered, but also intersect with other social categories such as age, race, and socioeconomic status, affecting individuals’ experiences of cyber violence in complex ways [34].

Older adolescents appeared to be more likely to witness cyber sex crimes, possibly due to higher degrees of exploration of online environments and increased awareness of such crimes. This could be due to increased online exploration and deeper engagement with different types of content. Additionally, it may be due to a better understanding of what cyber sex crimes entail and an enhanced awareness of how to recognize them.

Integrating the importance of voice and agency, it is critical to empower young women and girls to not only recognize but also to respond to and report cyber sex crimes. Empowering them to have a voice and agency in their online interactions aligns with the principles of feminist epistemology, which advocates for recognizing the voices of those most affected by cyber violence and supporting their ability to lead change in digital contexts [35]. By implementing educational programs that are sensitive to these intersectional identities and encouraging the active participation and leadership of young women and girls, we can foster a more inclusive and responsive approach to combating cyber sex crimes.

### 4.4. The Role of Teacher Intervention and the Understanding of the Systemic Nature of Cyber Sex Crimes

How adolescents perceive cyber sex crimes—whether they view them as problems resulting from systemic failures or as individual misdemeanors—significantly influences their reactions to these incidents. This underscores the importance of raising awareness about the systemic underpinnings of cyber violence, which may encourage more proactive attitudes among young people that emphasize the importance of collective responsibility and action. The findings from 2021 showed that teacher intervention did not directly prevent the witnessing of cyber sex crimes. However, this intervention played a critical role in helping adolescents understand that the recurrence of cyber sex crimes may be linked to systemic issues, such as insufficient legal sanctions that lack the severity to divert potential offenders or the inherent anonymity of the internet. Thus, teacher-led education and intervention have shown promise in helping adolescents understand the recurring nature of cyber sex crimes, despite the existing insufficient legal frameworks.

The crucial role of digital literacy in combating cyber sex crimes points to the need for educational programs to go beyond the purely technical aspects of online safety [17,25]. Rather, such programs should aim to foster an ethical and empathetic online culture. This includes teaching young people to critically evaluate online content. We argue that a deeper understanding among adolescents of how the recurrence of cyber sex crimes is linked to inadequate systemic responses could lead to increased attention to adopting proactive good citizenship behaviors, rather than attributing such actions solely to individual motivations for financial gain or attention-seeking. This study underscores the importance of a comprehensive educational strategy that not only addresses the mechanics of online safety but also cultivates a nuanced understanding of the motives behind cyber crime and how to perceive them.

### 4.5. Perceptions of Cyber Violence Influencing the Understanding of Cyber Sex Crimes

This study has shown that when adolescents perceive cyber violence as a critical issue in need of a solution, they are consequently more inclined to view cyber sex crimes as systemic problems. A feminist epistemology of digital crime highlights the importance of understanding how gender, power dynamics, and social norms influence these perceptions. It emphasizes that cyber sex crimes are not just criminal issues, but are deeply embedded in the patriarchal structures that shape digital interactions [34].

Educating adolescents about the seriousness of cyber sex crimes through this lens requires recognizing the gendered nature of these crimes and how they disproportionately affect women and marginalized groups. This understanding requires not only legal intervention but also a broader social movement and cultural shift to affect tangible change, echoing feminist calls for systemic reforms that address the root causes of gender-based violence in digital spaces [35].

Our results also show that knowledge of legal consequences is related to the idea that understanding cyber crime stems from individual motives. Thus, by raising awareness of the seriousness of these acts—while also integrating perspectives that recognize the intersectional impact of these crimes—adolescents will develop more sophisticated views on cybersex crime as a systemic issue. This may pave the way for a stronger collective call for systemic reforms aimed at more effectively addressing and preventing cyber sex crimes.

The lessons learned from this research provide a solid foundation for further research into effective strategies to combat cyber sex crimes and guide policy formulation. This emphasizes the critical integration of digital literacy, supportive interventions, and legal safeguards within educational and community settings. By incorporating feminist epistemology, these strategies can more effectively address the nuances of cyber violence, ensuring that interventions are inclusive and informed by an understanding of how cybercrimes intersect with issues of gender and power.

## 5. Conclusions

The study has explored the interplay between cyber violence and cyber sex crimes among adolescents in South Korea, a nation characterized by high digital engagement and early exposure to online platforms. Despite increased legal actions and public awareness of cyber sex crimes, we found that adolescents continue to experience cyber sex crimes. This suggests the need for a nuanced approach to combating these crimes, emphasizing not only the need for legal reforms but also profound shifts in societal attitudes and digital literacy.

Our research underscores the critical importance of digital literacy education so that adolescents can recognize, resist, and report cyber sex crimes and perceive cyber sex crimes as a systemic issue rather than an individual motivation.

A notable limitation of this study is the apparent underestimation of parental intervention. This may be due to the methodology used to measure the effectiveness of parental involvement. The measures of parental intervention were not directly related to educational discourse or discussions about cyber violence. Rather, they focused on regulating the behavioral aspects of adolescents’ internet use. In contrast, teacher interventions involved dialogues about cyber violence and actively encouraged adolescents to participate in these conversations. This distinction highlights the importance of voluntary engagement and intervention strategies that empower adolescents to think for themselves about the harmful effects of cyber violence and cyber sex crimes. Such findings suggest the need for approaches that not only address the manifestations of these issues but also engage youth in meaningful discussions that enhance their understanding of and responses to cyber violence.

## Figures and Tables

**Table 1:** Logistic regression predicting witnessing cyber sexual crime.

Year	Variables	*B*	*SE*	*Wald*	*p*	OR	95% CI OR
2021	Grade	0.06	0.02	15.24	<0.001	1.06	[1.03, 1.1]
	Gender	0.16	0.08	4.43	0.04	1.17	[1.01, 1.36]
	Average internet usage	0.00	0.03	0.00	0.98	1.00	[0.95, 1.05]
	Exposure to violent media content	0.15	0.01	233.90	<0.001	1.16	[1.14, 1.19]
	Experience of being a victim	0.36	0.09	16.78	<0.001	1.43	[1.2, 1.69]
	Experience of being a perpetrator	0.80	0.09	71.71	<0.001	2.22	[1.84, 2.66]
	Perception toward cyber violence	−0.06	0.08	0.60	0.44	0.94	[0.81, 1.09]
	Parent intervention	0.06	0.04	2.46	0.12	1.06	[0.99, 1.15]
	Teacher intervention	0.08	0.03	8.65	0.00	1.09	[1.03, 1.15]
	Viable response	−0.02	0.09	0.03	0.87	0.99	[0.83, 1.17]
	Legal consequences	0.06	0.09	0.40	0.53	1.06	[0.89, 1.26]
	Constant	−3.20	0.30	114.69	<0.001	0.04	
2022	Grade	−0.02	0.02	1.6	0.21	0.98	[0.94, 1.01]
	Gender	0.39	0.08	27.91	<0.001	1.48	[1.28, 1.72]
	Average internet usage	−0.01	0.03	0.12	0.73	0.99	[0.94, 1.04]
	Exposure to violent media content	0.18	0.01	363.70	<0.001	1.20	[1.17, 1.22]
	Experience of being a victim	0.43	0.08	28.19	<0.001	1.54	[1.31, 1.81]
	Experience of being a perpetrator	0.67	0.09	61.25	<0.001	1.96	[1.67, 2.33]
	Perception toward cyber violence	−0.23	0.07	12.36	<0.001	0.79	[0.7, 0.90]
	Parent intervention	0.06	0.04	3.04	0.08	1.07	[0.99, 1.14]
	Teacher intervention	0.03	0.03	1.26	0.26	1.03	[0.98, 1.08]
	Viable response	−0.11	0.07	2.16	0.14	0.90	[0.78, 1.04]
	Legal consequences	0.07	0.08	0.82	0.37	1.07	[0.92, 1.24]
	Constant	−2.12	0.27	62.31	<0.001	0.12	

**Table 2:** Logistic regression on perceiving cyber sex crime as a systemic issue.

Year	Variables	*B*	*SE*	*Wald*	*p*	OR	95% CI OR
2021	Grade	0.04	0.01	19.14	<0.001	1.04	[1.02, 1.06]
	Gender	0.23	0.04	27.41	<0.001	1.26	[1.15, 1.37]
	Average internet usage	0.09	0.02	38.07	<0.001	1.10	[1.06, 1.13]
	Exposure to violent media content	−0.02	0.01	19.08	<0.001	0.98	[0.97, 0.99]
	Experience of being a victim	0.05	0.06	0.91	0.34	1.05	[0.95, 1.18]
	Experience of being a perpetrator	−0.14	0.07	3.84	0.05	0.87	[0.76, 1.00]
	Perception toward cyber violence	0.33	0.05	54.11	<0.001	1.39	[1.27, 1.52]
	Parent intervention	0.02	0.02	0.82	0.37	1.02	[0.98, 1.06]
	Teacher intervention	0.03	0.02	4.41	0.04	1.03	[1.00, 1.07]
	Viable response	−0.01	0.05	0.01	0.91	0.99	[0.90, 1.10]
	Legal consequences	−0.16	0.05	9.99	0.00	0.85	[0.77, 0.94]
	Witnessing cyber sex crime	0.01	0.08	0.03	0.86	1.01	[0.87, 1.18]
	Cyber violence education necessity	0.15	0.04	17.68	<0.001	1.16	[1.08, 1.25]
	Constant	−2.68	0.20	189.84	<0.001	0.07	
2022	Grade	0.04	0.01	18.38	<0.001	1.04	[1.02, 1.06]
	Gender	0.35	0.04	66.51	<0.001	1.42	[1.30, 1.54]
	Average internet usage	0.02	0.02	1.41	0.24	1.02	[0.99, 1.05]
	Exposure to violent media content	0.00	0.01	0.36	0.55	1.00	[0.99, 1.02]
	Experience of being a victim	0.08	0.05	2.48	0.12	1.08	[0.98, 1.19]
	Experience of being a perpetrator	−0.03	0.06	0.19	0.67	0.98	[0.87, 1.09]
	Perception toward cyber violence	0.14	0.04	12.38	<0.001	1.14	[1.06, 1.23]
	Parent intervention	0.01	0.02	0.08	0.78	1.01	[0.97, 1.05]
	Teacher intervention	0.03	0.01	5.26	0.02	1.03	[1.01, 1.06]
	Viable response	0.01	0.04	0.04	0.85	1.01	[0.93, 1.10]
	Legal consequences	−0.07	0.04	2.86	0.09	0.93	[0.85, 1.01]
	Witnessing cyber sex crime	−0.05	0.07	0.46	0.50	0.95	[0.83, 1.10]
	Cyber violence education necessity	0.12	0.03	14.92	<0.001	1.12	[1.06, 1.19]
	Constant	−1.46	0.17	71.38	<0.001	0.23	

## Data Availability

Restrictions apply to the availability of these data. Data were obtained from Korea Communications Commission (KCC) and are available from the authors upon request with the permission of Korea Communications Commission (KCC). The data collection was managed by the Korea Communications Commission (KCC) and the National Information Society Agency (NIA).

## Appendix A

**Table A1:** Correlation table for 2021.

	Variable	n	M	SD	1	2	3	4	5	6	7	8	9	10	11	12	13
2021	Grade	9016	4.99	2.567	1												
	Gender	9016	0.5	0.5	−0.046 **	1											
	Average internet usage	9016	3.39	1.518	0.137 **	0.026 *	1										
	Exposure to violent media content	9016	−0.138846	4.2511652	0.115 **	−0.033 **	0.056 **	1									
	Experience of being a victim	9016	0.22	0.416	−0.072 **	−0.003	0.062 **	0.139 **	1								
	Experience of being a perpetrator	9016	0.14	0.343	−0.055 **	−0.041 **	0.052 **	0.155 **	0.326 **	1							
	Perception toward cyber violence	9016	3.61915	0.538732	0.008	0.055 **	0.047 **	−0.114 **	−0.002	−0.080 **	1						
	Parent intervention	9016	1.3425	1.12553	−0.253 **	0.027 *	−0.172 **	0.035 **	−0.004	−0.01	0.066 **	1					
	Teacher intervention	9016	2.3016	1.53903	−0.128 **	0.02	−0.026 *	−0.051 **	−0.025 *	−0.041 **	0.170 **	0.404 **	1				
	Viable response	9016	0.44	0.496	−0.051 **	0.005	0.027 *	−0.085 **	−0.012	−0.017	−0.001	0.068 **	0.153 **	1			
	Legal consequences	9016	0.34	0.475	0	−0.041 **	−0.002	−0.066 **	−0.030 **	0.007	−0.038 **	0.057 **	0.108 **	0.445 **	1		
	Witnessing cyber sex crime	9016	0.09	0.287	0.043 **	0.01	0.017	0.193 **	0.098 **	0.142 **	−0.025 *	0.027 **	0.022 *	−0.009	−0.001	1	
	Cyber violence education necessity	9016	3.4584	0.65354	−0.105 **	0.060 **	−0.027 *	−0.081 **	−0.016	−0.042 **	0.258 **	0.158 **	0.233 **	0.044 **	0.029 **	−0.02	1

* *p* < 0.05, ** *p* < 0.01

**Table A2:** Correlation table for 2022.

	Variable	n	M	SD	1	2	3	4	5	6	7	8	9	10	**11**	**12**	**13**
2022	Grade	9689	4.77	2.531	1												
	Gender	9689	0.5	0.5	0.037 **	1											
	Average internet usage	9689	3.7	1.567	0.346 **	0.087 **	1										
	Exposure to violent media content	9689	−0.000774	4.0399043	0.395 **	0.035 **	0.277 **	1									
	Experience of being a victim	9689	0.37	0.483	−0.064 **	−0.111 **	0.098 **	0.224 **	1								
	Experience of being a perpetrator	9689	0.21	0.404	−0.069 **	−0.164 **	0.105 **	0.180 **	0.446 **	1							
	Perception toward cyber violence	9689	3.65988	0.574348	0.042 **	0.139 **	0.026 *	0.076 **	−0.015	−0.073 **	1						
	Parent intervention	9689	1.0774	1.10811	−0.305 **	0.069 **	−0.256 **	−0.121 **	−0.034 **	−0.035 **	0.098 **	1					
	Teacher intervention	9689	2.3945	1.61512	−0.247 **	0.078 **	−0.109 **	−0.053 **	−0.064 **	−0.029 **	0.170 **	0.376 **	1				
	Viable response	9689	0.55	0.498	−0.090 **	−0.011	−0.066 **	−0.074 **	0.034 **	0.023 *	0.031 **	0.119 **	0.155 **	1			
	Legal consequences	9689	0.56	0.497	0.018	−0.008	−0.029 **	0.034 **	−0.001	0.020 *	0.055 **	0.126 **	0.166 **	0.242 **	1		
	Witnessing cyber sex crime	9689	0.1	0.302	0.054 **	0.030 **	0.067 **	0.254 **	0.143 **	0.159 **	−0.016	−0.006	0.001	−0.02	0.019	1	
	Cyber violence education necessity	9689	3.4689	0.73984	−0.159 **	0.136 **	−0.086 **	−0.101 **	−0.084 **	−0.096 **	0.223 **	0.179 **	0.260 **	0.098 **	0.085 **	−0.055 **	1

* *p* < 0.05, ** *p* < 0.01
